# Experimental and Environmental Analysis of High-Strength Geopolymer Based on Waste Bricks and Blast Furnace Slag

**DOI:** 10.3390/polym15143092

**Published:** 2023-07-19

**Authors:** Jan Fořt, Martin Mildner, Martin Keppert, Vojtěch Pommer, Robert Černý

**Affiliations:** Department of Materials Engineering and Chemistry, Faculty of Civil Engineering, Czech Technical University in Prague, Thakurova 7, 166 29 Prague, Czech Republic; martin.mildner@fsv.cvut.cz (M.M.); martin.keppert@fsv.cvut.cz (M.K.); vojtech.pommer@fsv.cvut.cz (V.P.); cernyr@fsv.cvut.cz (R.Č.)

**Keywords:** geopolymer, alkali activation, brick powder, blast furnace slag, mechanical strength, environmental footprint

## Abstract

The rationalization of material flows, together with the utilization of waste raw materials for the production of alternative binders, became a very attractive topic during the last decades. However, the majority of designed materials can be used as a replacement for low-performance products. In this work, the waste materials (brick powder and blast furnace slag) are valorized through geopolymerization to design high-performance material as an alternative to high-performance concrete. Designed mixtures activated by sodium silicate and waste-originated alkali solution are characterized by the meaning of the chemical and mineralogical composition, evolution of hydration heat, and mechanical strength test. To contribute to the understanding of the environmental consequences and potential benefits, the carbon footprint and embodied energy analysis are provided. Obtained results highlight the potential of end-of-life bricks for the design of high-performance composites if mixed together with more reactive precursors. Here, even values over 60 MPa in compressive strength can be achieved with the dominant share of low-amorphous brick powder. The higher crystalline portion of brick powder may lead to the reduction of drying shrinkage and preservation of flexural strength to a greater extent compared to used slag. Performed environmental analysis confirmed the CO_2_ emission savings; however, the embodied energy analysis revealed a huge impact of using alkaline activators.

## 1. Introduction

Since the exorbitant depletion of natural resources and the generation of huge amounts of waste negatively affects the stability of ecosystems and creates a significant threat to future human well-being, rethinking the materials base represents an important challenge for present society [1]. Namely, about 4.8 tons of waste are produced per EU inhabitant, while only 39.2% of waste is recycled. This issue is relevant particularly in the construction industry as it produces the largest amount of waste across all industrial fields (EU—37.5%). Apart from the concrete, bricks, and ceramic waste stand for approx. 45% of the total amount of construction and demolition waste (CDW) [2,3]. Taking into account the service life of the buildings, the generation of brick-based waste is accelerating and creates a challenge for efficient the destination of this waste to avoid undesired landfilling and adverse effects on the natural environment [4]. Speaking in terms of numbers, about 800 million tons of CDW were produced in the EU in 2020, with an increasing trend, and thanks to rapidly increasing urbanization, an increase of about 200% is expected by 2050. In line with urbanism growth, the production of building materials generates a huge environmental footprint, including greenhouse gas emissions [5].

The development of sophisticated structures in this respect places higher demands on mechanical properties, and the use of high-performance concrete (HPC) or ultra-high-performance concrete (UHPC) is becoming more and more popular [6]. However, such materials require high dosages of energy-intensive and costly materials such as Portland cement, silica fume, and superplasticizers. For example, the dosage of Portland cement for HPC exceeds 700 kg/m^3^, thus noticeably shifting the environmental footprint compared to conventional concrete [7]. To optimize the composition of high-strength concrete and reduce cost and environmental burden, various initiatives can be recognized in the literature [8]. In general, the use of fly ash (FA) or granulated blast furnace slag (GBFS) was found as an efficient approach that allows the replacement of up to 40–50% of cement weight and concurrently provides the desired level of mechanical performance [9]. Yu et al. [10] achieved an increase in the compressive strength of about 10% if 30% of Portland cement was alternated by GBFS. Liu and Guo [11] reported on the adverse effects of supplementary cementitious materials (SCMs) used as cement replacement on lowered early-age strength as a result of reduced reactivity compared to Portland cement. The limited reaction kinetics was also assumed by Yang et al. [12] due to negative effects associated with low Al_2_O_3_ content, and increased P_2_O_5_ content may result in retardation of the hydration mechanism and strength increase. Similar effects on strength development were reported for using fly ash by Wu et al. [13], who reached about an 8% reduction in compressive strength by the replacement of 13.5 wt.% of Portland cement. FA usually degrades the strength of modified high-performance composites; however, in selected papers, mechanical strength benefits are recognized, and the main advantages are rather related to cost and environmental savings [8].

In this respect, the replacement of raw materials and utilization of waste take place to mitigate undesired consequences on the natural environment, ecosystems, and human health. Specifically, strategies based on cement replacement or natural aggregate replacement can be identified with a focus on a broad range of supplementary cementitious materials, including alkali activation [14]. The process of alkali activation, consisting of the dissolution of precursor (FA, GBFS, metakaolin, etc.) by the alkaline activator (usually a mix of sodium hydroxide and sodium silicate), results in polycondensation and re-polymerization reactions and the formation of dense materials with desired functional performance (alkali-activated materials-AAMs). These materials can be divided into two general groups: Ca-rich AAMs formed by C-A-S-H gel and N-A-S-H gel, also called geopolymers [15]. In the literature, can be found many differences between these two groups, and these concepts are also often not distinguished clearly. Such formed materials can find employment in various applications due to their resistance to aggressive environments, sufficient mechanical strength, and lowered environmental footprint. Significant effort was paid to the understanding of the use of fly ash, blast furnace slag, metakaolin, clays, zeolites, and brick powder [16,17,18]. Nonetheless, the vast majority of available studies remain aimed at lowering the environmental impact and reaching only satisfactory performance to replace normal-strength concrete [19]. For example, Reig et al. [20] prepared metakaolin-based AAM with a maximum compressive strength of about 50 MPa despite an increased level of material porosity compared to conventional concrete. The study by Hwang et al. [21] describes the compressive strength from 36 MPa to 70 MPa at ambient curing temperature using waste brick powder (WBP) as a primary precursor. However, the WBP needed to be supplemented by FA or GBFS as the pure WBP was not able to set and harden. The SEM energy dispersive spectroscopy analysis revealed the correlation between Si/Al ratio and compressive strength after 28 days of curing. It was found that the application of FA decreased the reaction kinetics and amount of dissolved Ca^2+^ and consequently affected the formation of sodium aluminate silicate hydrate gel with lower strength compared to calcium aluminate silicate or calcium silicate hydrate gel. On the contrary, higher CaO content in GBFS/WBP mixture showed a denser structure of formed C-A-S-H gel that has a beneficial effect on compressive strength [22]. The best performance of blended precursor based on WBP and GGBFS was achieved by [23], who reached a compressive strength of around 100 MPa using sodium carbonate or sodium hydroxide. However, they were not able to form a dense structure for pure WBP and significant portions of GBFS were required.

Similar numbers can be found in many other research reports using a broad range of precursors. Besides the effect of applied precursors, the curing condition and dosage of applied activators were studied as well [24]. Despite the wide range of studied precursors, further research is required to provide a coherent understanding of the hardening mechanism and reach the desired strength level [25].

Besides the functional performance, significant attention is paid to potential benefits related to the environmental performance of AAMs, as the omitted consumption of Portland cement, together with the use of waste materials, reduces energy intensity [26,27]. On the other hand, applying sodium silicate or sodium hydroxide is associated with noticeable emissions and damages the environmental footprint. Available studies assume the potential environmental saving between 20–80% according to used materials, curing type, and used assessment methodology [4]. These findings may be relevant, especially with the connection to the energy intensity of HPC with increased demand for the consumption of energy-intensive constituents. However, such research is missing despite its particular importance in this field.

Considering the above-revealed gaps in the available literature, the main aim of this study is to highlight the potential of waste bricks as a suitable precursor for alkali activation that may help close loop recycling of CDW and significantly contributes to lowering the environmental profile of the building industry. The utilization of waste brick powder as an end-of-life material promotes the circularity of material flows in the building industry and can be accepted as an exemplary demonstration of CDW upcycling despite the lower reactivity. To boost the mechanical strength, the performed study deals with the utilization of waste brick powder together with GBFS to obtain high-performance material. Six mixtures were designed, including pure WBP and GBFS mixes and their combinations in ratios of 80:20; 60:40; 40:60, and 20:80. Consequently, the understanding of the geopolymerization and the hardening mechanism is done by the employment of X-ray diffraction and fluorescence analysis supplemented by isothermal calorimetry. The determination of mechanical properties is done, and obtained results are linked together with carbon dioxide emissions related to the production of input materials to access the combined functional and environmental performance. The obtained results refer to the potential of waste bricks and the potential valorization of this abundant material.

## 2. Materials and Methods

### 2.1. Used Materials

**Figure 1:**
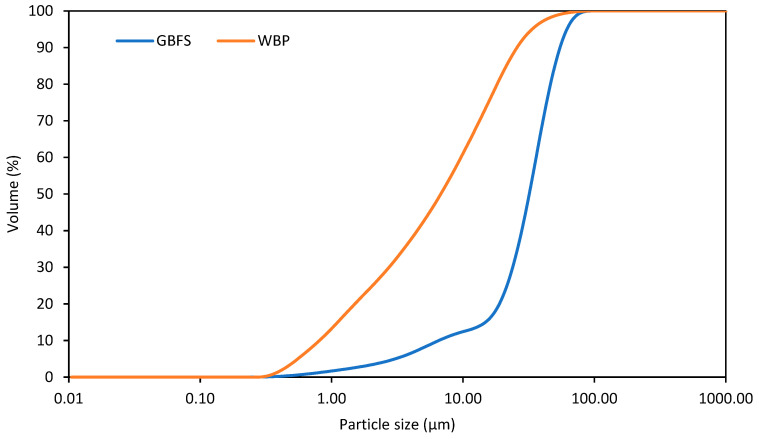
Particle size distribution of GBFS and WBP.

The waste brick powder originated from end-of-life red bricks (around 100 years old) and was collected on a landfill site. Collected bricks were crushed in a jaw crusher and milled in a ball mill (KM 01/R, Artik studio, Ústí nad Labem, Czech Republic) to reach the desired fineness. Then, the dried brick powder was sieved in order to separate larger particles or shards and obtain a homogenous material. Only particles smaller than 0.063 mm were alkali activated because of their better reactivity promoting the mechanical performance of designed materials in the hardened state. The collected brick powder was dried at 80 °C for 48 h to remove redundant moisture. The granulated blast furnace slag was purchased from Kotouč Štramberk as a secondary precursor to supplement the composition of WBP due to its desired composition based on the CaO–SiO_2_–MgO–Al_2_O_3_ system. The particle size distribution of both precursors is provided in Figure 1.

The X-ray diffraction results (see Table 1) were evaluated using a Malvern PANanalytical Aeris device (Malvern, Great Britain) with the HighScorePlus software package (version 3.0.5) and JCP DS PDF2 database. The Rietveld analysis was done with TOPAS software to quantify the amorphous portion (by using an internal standard (ZnO, 10%). The oxide composition of the materials was characterized by X-ray fluorescence spectroscopy analysis (device ARL 9400 XP, Thermofisher Scientific, Waltham, MA, USA), and results are provided in Table 2. As shown, a dominant part is composed of SiO_2_ and CaO with a minor part of Al_2_O_3_ and MgO.

For the preparation of a suitable alkaline activator, a combination of waste sodium hydroxide from the glass industry and sodium silicate (water glass) with SiO_2_/Na_2_O molar ratio of 1.6 (Vodní sklo, a.s., Brno-jih, Czech Republic) was applied.

The slag is, in terms of phase composition, a highly glassy material containing just a small amount of akermanite and calcite. In trace amounts, quartz and merwinite are also present. The brick powder contains a high amount of quartz, accompanied by several feldspars (anorthite, microcline, orthoclase) and mica (probably muscovite). The amorphous portion of brick is just 30%; it is a material having its origin in fired clay minerals used for brick production.

Geopolymers were prepared through the alkali activation of GBFS and WBP by the mixture of sodium silicate and waste activator (WA) based on sodium hydroxide [28]. The detailed composition, together with mixture labeling, is provided in Table 3. The last column specifies the Si/Al ratio of the precursor. The content of water was slightly modified to obtain the material with the same rheologic properties (spread diameter = 140 mm). First, the GBFS and WBP were blended together to obtain a homogenous mixture. The activation solution was prepared from solid WA dissolved in water and water glass in liquid form. The solution thus prepared was poured into the mixture of precursors and first mixed by hand. The mixture was then mixed using an automatic mixer for 60 s at low speed and 30 s at high speed. After that, the mixing was stopped, and the mortar that stuck to the walls or the bottom of the bowl was wiped off with the help of a trowel. The procedure was finished with 60 s of high-speed mixing. Three prismatic samples with dimensions of 40 mm × 40 mm × 160 mm were cast for each mixture and stored for 28 days in a highly humid environment (90 ± 5%) at a constant temperature (21 °C).

### 2.2. Employed Experimental Methods

An Analysette 22 MicroTec plus (Fritsch, Idar-Oberstein, Germany) laser diffraction device with a measuring range of up to 2 mm was used for the determination of the particle size distribution of the applied waste brick powder.

A TAM Air eight-channel calorimeter (TA Instruments, New Castle, DE, USA) was used for monitoring the development of reaction heat of prepared geopolymers. Each calorimetric channel is a twin type, consisting of a reference chamber and a sample. The ampoules of the calorimeter have a volume of 20 mL. The operating temperature range is 5–90 °C, and the detection limit is 4 W.

Scanning electron microscopy (SEM) was used to analyze the microstructure of designed geopolymers. The SEM images were obtained by a TESCAN MIRA3 XMU device (Tescan, Brno, Czech Republic) on thin slices of materials cut by using a precision saw. Samples were cut into slight slices and subjected to SEM analysis. The mercury intrusion porosimetry (MIP) was for the characterization of the pore size distribution. Here, a combination of Pascal 140 and Pascal 440 porosimetry (Thermo Fisher Scientific, Waltham, MA, USA) was used to provide a coherent description of the porous space. 

The bulk density, matrix density, and total open porosity were determined by the weighting of the samples with known volume and by a helium pycnometer Pycnomatic ATC (Thermo Fisher Scientific, Waltham, MA, USA). A hydraulic testing device VEB WPM Leipzig with a stiff loading frame with a capacity of 3000 kN, was used for the measurement of compressive and flexural strength. Three prismatic samples having dimensions of 40 mm × 40 mm × 160 mm were used for each set of samples. The compressive strength was measured on the prisms broken in the flexural test; the loading area was 40 mm × 40 mm. The loading speed within the experiment was 0.6 MPa/s.

### 2.3. Carbon Dioxide Emissions Assessment

The environmental performance of designed mixtures is determined in order to provide a comparison to mixtures having similar mechanical performance. Therefore, the environmental impact analysis of used input materials is carried out with an emphasis on carbon dioxide emissions. One cubic meter of designed paste with compressive strength of about 60 MPa is considered a functional unit, so material and emission flow related to its production can be included in the scope of the analysis. The emission data and consumed energy necessary for the inventory were compiled from the literature review [28,29,30,31], theoretical estimations, and Ecoinvent database v3.4 by using SimaPro SW 9.0.

The impact categories of cumulated energy consumption and carbon dioxide emissions were taken into account during the environmental analysis. The carbon dioxide emissions were stated in kg CO_2_ equivalent, and the particular importance of gases with the most harmful effect on global warming was included. Consequently, the input energy efficiency, as well as carbon dioxide intensity, were combined with functional performance to access the comparable indicator of environmental score per functional unit [29]. The energy efficiency was calculated based on the following formula:

(1) $$Ei=\frac{e}{p}$$

where *e* (MJ) is the energy related to the production of a functional unit of the binder, and *p* is the compressive strength *R_c_* (MPa). 

The carbon dioxide intensity was retrieved from the following equation:

(2) $$Ci=\frac{c}{p}$$

where *c* (kg CO_2_) is the total CO_2eq_ emission related to the functional unit.

## 3. Results and Discussion

### 3.1. Phase Composition and SEM Analysis

**Figure 2:**
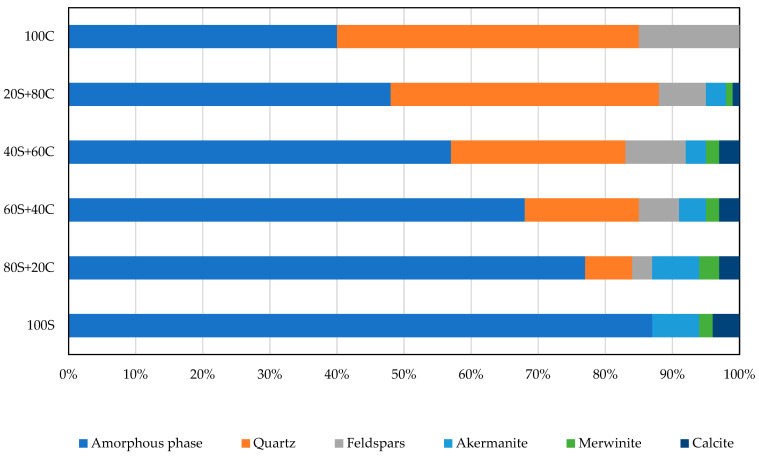
Phase composition of activated products.

The XRD analysis of formed products provides an important insight into the mineralogical composition. The phase composition of prepared geopolymers more or less linearly corresponds to the composition of mixed precursors (Figure 2). The activated brick (100C) contains 40% of amorphous matter, while the rest is quartz and feldspars from the raw brick. As shown, the portion of the amorphous phase was increased in comparison to the original WBP precursor. The mica diffractions disappeared during alkaline activation. As the content of slag in the mixed precursor has been growing, the content of quartz and feldspars in activated products decreased and, obviously, increased the content of minerals related to slag (akermanite, merwinite, and calcite). Contrary to results reported in [23], no content of muscovite, hematite, or faujasite was revealed. This finding can be assigned to different compositions of used precursors. On the other hand, the determination of phase composition provides a more detailed understanding of formed products and their relation to functional properties.

**Figure 3:**
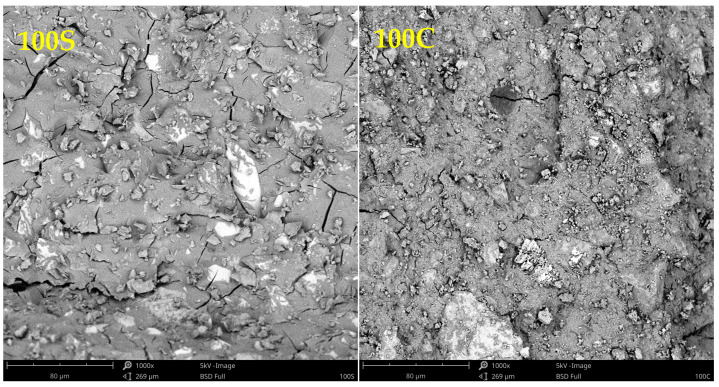
SEM images of 100S and 100C mixture.

Figure 3 shows differences between the mixture composed of GBFS and WBP only. Here, one can see a number of cracks in the mixture based on GBFS, while the mixture with WPC does not exhibit the same extent of crack occurrence. As the drying shrinkage is accompanied by the high reactivity of the precursor, the obtained findings comply with results from other performed experiments [28]. 

### 3.2. Isothermal Calorimetry

**Figure 4:**
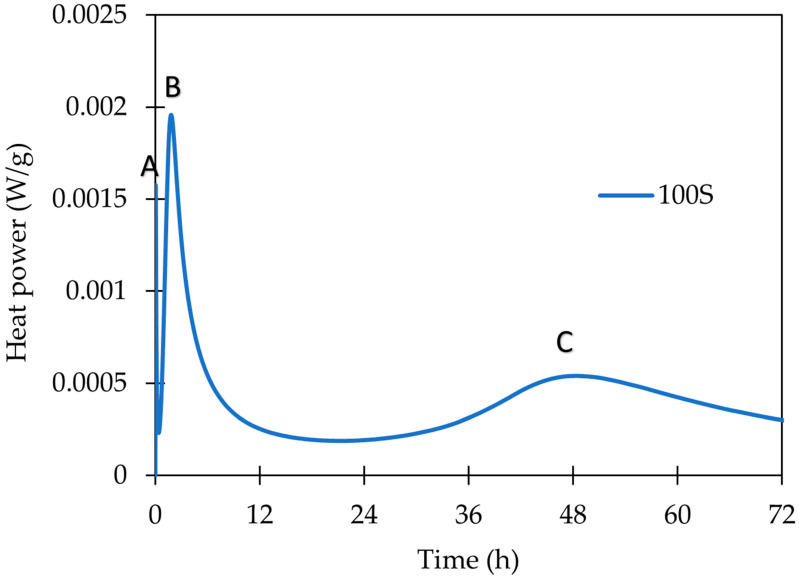
Time development of heat power during the first 3 days of polymerization of the S100 sample.

The isothermal (20 °C) calorimetry experiment was performed in order to compare the reaction course of different mixed precursors. Generally, the alkaline activation of an aluminosilicate precursor may comprise up to three exothermic processes observable by the isothermal calorimetry. All of these three processes are clearly observable in the case of alkaline activation of the pure slag sample 100S (Figure 4). The first exothermic peak (A) starts to develop immediately after the mixing of the precursor and activator. This heat generation is due to the alkaline hydrolysis of the precursor, which dissolves into fragments serving as monomers for the geopolymerization. This step features a maximum heat flow after a few minutes. The second exothermic peak (B) is due to the polycondensation of monomers from precursors to oligomers, and the third one (C) is interpreted as a “rearrangement” of the materials structure of formed products or as polymerization of oligomers to polymers.

**Figure 5:**
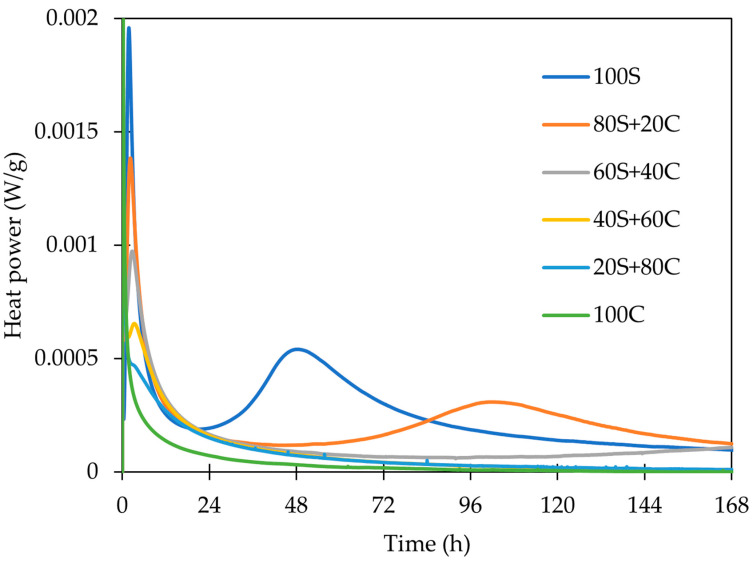
The entire hydration course of alkali-activated materials.

**Figure 6:**
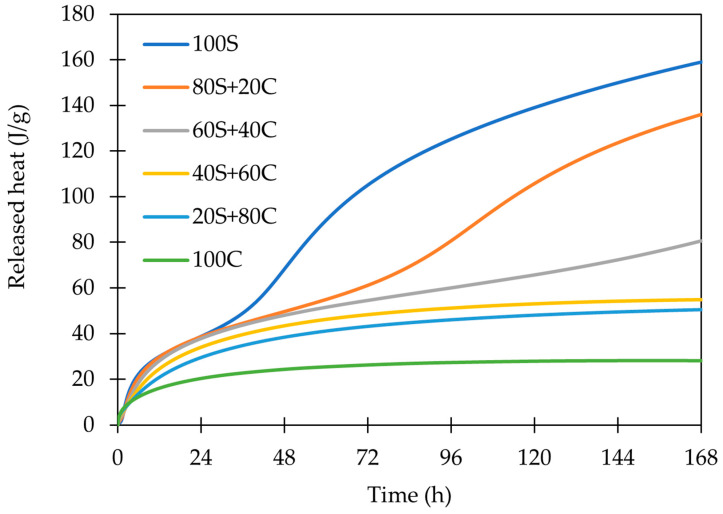
The entire cumulative hydration course of alkali-activated materials.

The comparison of all designed mixtures depicted in Figure 5 and Figure 6 shows a clear trend from 100S to 100C (activated brick): GBFS (100S) provides high reaction heat; brick is not just “diluting” the system and reducing the total heat evolved, but it is also shifting the peak C to the higher time. The heat development of activated pure brick (100C) does not show any signals B and C. It does not necessarily mean that the geopolymerization process is not taking place in this system—it does, but apparently, it is not as exothermic and intensive as in slag. Moreover, the role of low-amorphous precursors is still not fully elucidated in this sense and requires further research. The cumulative heat release can be further increased by the addition of the higher activator dosage as a relationship between Na_2_O influences the reaction degree [4]. A similar observation was also noted by Najimi et al. [32], who attributed the higher reactivity of GBFS to CaO content. The geopolymeration rate corresponds to the differences in the amorphous content for both used precursors. It should be noted that the amorphous phase portion in the WBP precursor was lower compared to the formed geopolymer. 

**Figure 7:**
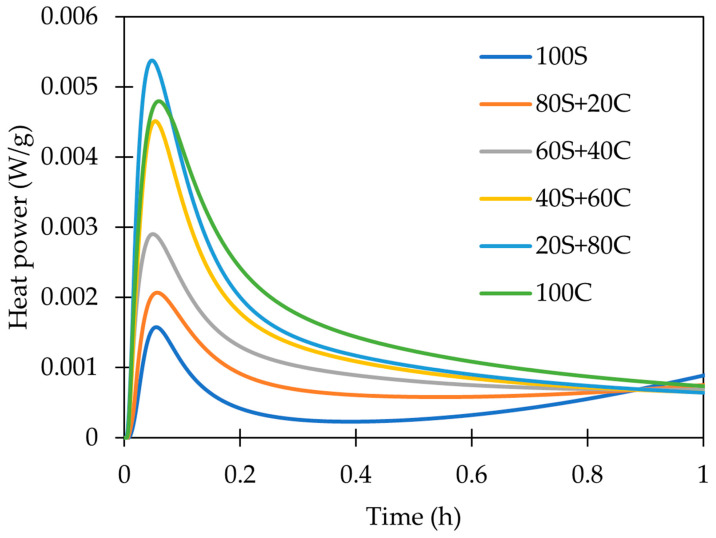
The first hour of hydration (peak A) in alkali-activated systems.

On the other hand, signal A—the dissolution of precursor—is much more intensive in brick than in the GBFS (Figure 7). It might imply that in the case of brick-rich samples, all of the steps (dissolution, polycondensation, rearrangement) are taking place very quickly with just the single peak A. One has to also keep in mind that the brick precursor is highly “diluted” by quartz and feldspars, what is lowering the specific heat of occurring processes. The presented outcomes comply with the observation of Shen et al. [33], who assigned the first initial peak to the rapid dissolution of brick particles.

### 3.3. Basic Materials Properties

**Figure 8:**
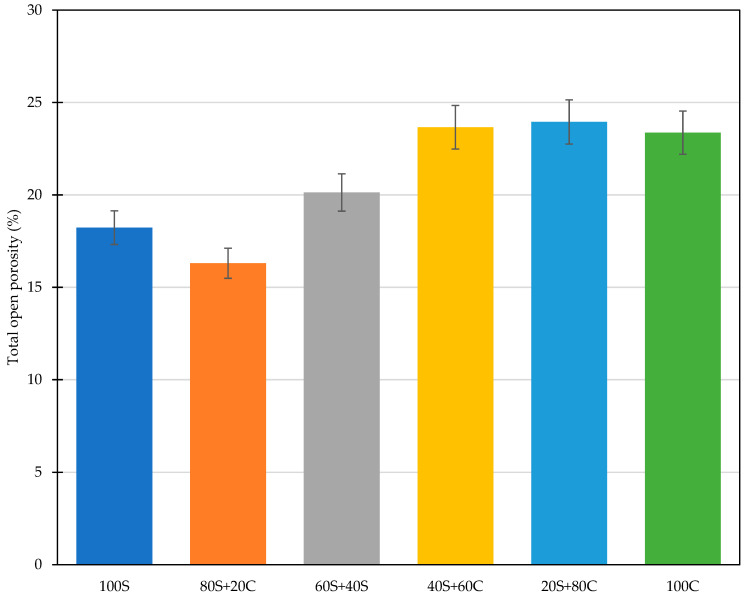
Total open porosity of designed mixtures.

Basic materials properties in terms of bulk density, matrix density, and total open porosity are presented in Table 4 and Figure 8. Here, only small variations can be recognized. The most compact structure was obtained for the mixture based on GBFS only, while a further increase in the WBP content lowered the bulk density and therefore increased the porosity of the designed samples. The compactness of the microstructure relies on the intensity of the geopolymerization and reactivity of used precursors. 

### 3.4. MIP Analysis

**Figure 9:**
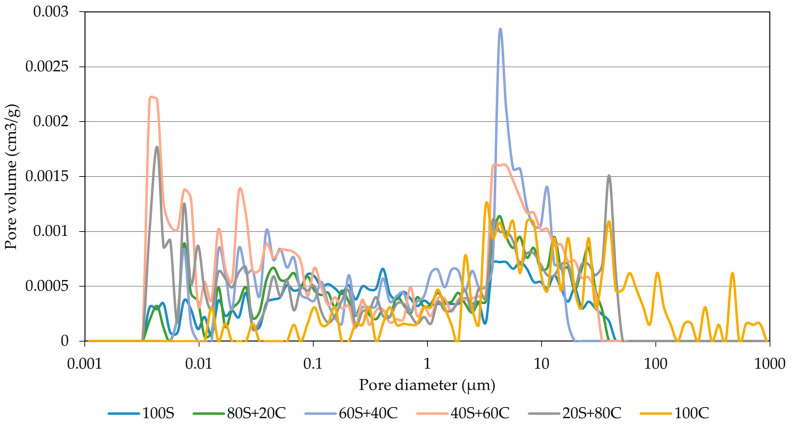
Pore size distribution.

The pore size distribution curves of designed mixtures are plotted in Figure 9. As can be seen, significant differences between particular mixtures can be observed. Specifically, the mixture prepared only by GBFS revealed the lowest level of pore volume in almost all size intervals. A similar performance was achieved by mixtures with dominant GBFS content (up to 40 wt.%). Mix 60S40C led to an increase of pores in the area around 10 µm. The increase in the ratio of brick to slag led to significant changes in the microstructure of the material. Namely, the mix with pure brick powder precursor exhibits a notable shift in the range from 100 to 1000 µm. The mixtures with 60 and 80 wt.% of brick powder resulted in a substantial increase in the range of 0.005 µm and 0.1 µm. On the other hand, the content of small pores for pure WBP was significantly reduced. This phenomenon can be linked to the formation of a denser structure and the filling of the micropores. The beneficial effect of brick particles on lowering average pore size was described by Sedira et al. [34], who used brick powder together with tungsten mining waste. The obtained results clearly correspond to the results of the basic material properties elucidated in the previous paragraph and comply with the previous findings of Soultana et al. [35]. However, the lower reactivity and higher crystalline part content of WBP diminished the benefits of fine WBP particles, and further efforts aimed at improvement of WBP reactivity should be performed [36]. It should be highlighted that the increase in the larger pores range is associated with the reduction in the mechanical strength. On the other hand, the widely accepted relationship between porosity and compressive strength cannot be applied to the same extent as for cement-based materials due to the broad range of formed reaction products with different mechanical strengths. The changes in the pore size formation refer to the importance of molar ratios (Si/Al; Na/Si+Al), also as described in the work of Li et al. [37]. In this sense, the porous space can be modulated by the adjustment of alkaline activator dosages or concentration. Specifically, the increase in the dosage (Na/Si+Al) ratio led to a shift in the transition pores (0.01–0.1 µm) and the simultaneous formation of larger pores due to the sensitivity of WBP on the concentration of the applied activator. It also correlates with the increased amount of feldspar that appeared in the 100C mix.

### 3.5. Mechanical Properties

**Figure 10:**
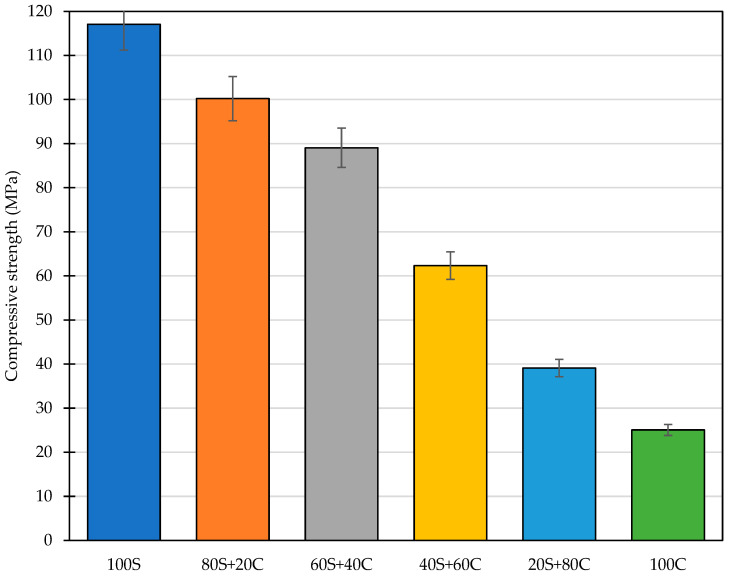
Compressive strength of designed mixtures.

**Figure 11:**
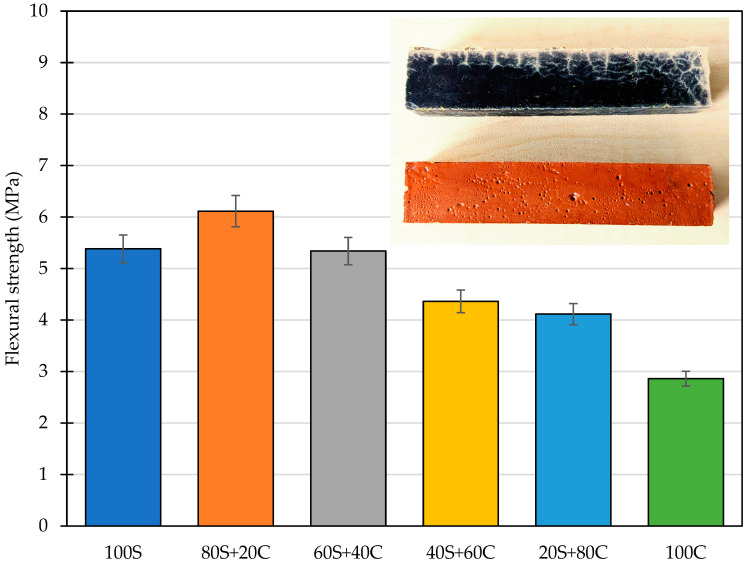
Flexural strength of designed mixtures.

The mechanical results determined after 28 days of normal conditions curing are given in Figure 10 and Figure 11. As shown, the compressive strength was gradually decreased in line with the increasing content of waste brick powder from 117 MPa obtained for pure GBFS precursor. On the other hand, even a mixture with 100% of WBP formed a dense structure with sufficient mechanical strength, contrary to the conclusions of Rakhimova and Rakhimov [23] or Hwang et al. [21], who failed in the polymerization of the brick powder through alkali activation. The applicability of WBP as a precursor was previously confirmed in several studies [29,38], where the potential of WBP was described. The compressive strength was gradually reduced from 117 MPa (pure GBFS) to approx. 25 MPa (pure WBP), and even the mix with 60 wt.% of WBP exceeded 60 MPa in compressive strength. The apparent reason for the capability of pure WBP to form a dense structure can be found in the mixture composition and the role of the molar ratios in particular. In the work of Hwang et al. [21], a higher dosage of NaOH shifted the Na/Si ratio, which may lead to the depolymerization of reaction products, and weakening of the microstructure as a result of the interaction of Na^+^ ions with Si-OH and Al-OH [39].

The obtained trend in the reduction in the compressive strength corresponds well to other research papers where similar constituents were applied. The results of Rakhimova and Rakhimov [23] point to the importance of materials preparation since the conjoint milling of both precursors provides better mechanical performance over separate milling. The benefits of conjoint milling were confirmed by the increase in the density and lower material porosity, which refers to a better reactivity of blended precursor. Together with the improvement in the mechanical strength related to increased temperature curing up to 90 °C [40], a significant shift in the functional performance can be obtained supported by the advanced mixture design optimization techniques [41]. 

The flexural strength was, contrary to previously discussed papers, determined due to its importance for the understanding of diverse mechanical parameters compared to concrete and to highlight another weak point of alkali-activated materials. The flexural strength of designed mixtures did not exhibit such significant reduction as observed for the compressive strength, namely the initial flexural strength obtained for 100 S was reduced from 5.4 MPa to 2.9 MPa, while the highest performance was achieved by the 80S+20C mixture, which exceeded 6 MPa. Such values, relatively low in comparison to HPC, refer to the substantial impact of the shrinkage and can be associated with the dosage of GBFS. Although some authors neglect the complications with the drying shrinkage occurrence, the study of Lin et al. [42] shows the particular importance of mix proportioning, activator dosage, and CaO content. They concluded that the higher GBFS dosage may lead to worsening the shrinkage and consequently negatively affect the durability properties, the flexural strength in particular due to increased brittleness of samples. This finding complies with the higher hydration heat evolution as well as the change in the composition provided by XRD analysis. Namely, the increase in the content of quartz (crystalline) acts as a filler and mitigates the drying shrinkage. A dual role of the low amorphous precursors and their contribution to the mechanical performance was characterized previously by Fořt et al. [40].

Further improvements in mechanical performance can be made through the employment of thermodynamic modeling that allows the advanced approach for the strength prediction based on the chemical and mineralogical composition of the used precursors, reaction kinetics affected by the type of applied activators, and phase assemblages. It may significantly help the understanding of the correlation between the chemical composition of precursors and functional parameters [43,44]. 

### 3.6. Carbon Dioxide Emissions

**Figure 12:**
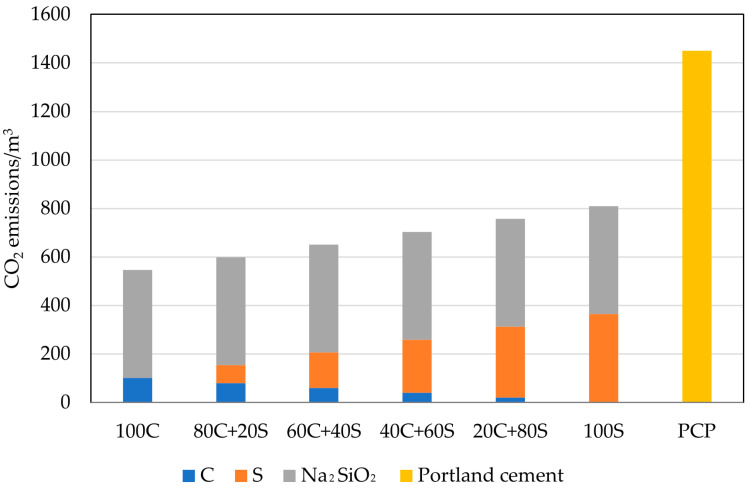
Comparison of the carbon dioxide footprint.

The main driving factor of geopolymer research consists in the lowered environmental footprint of such materials, reduced CO_2_ emissions, and energy consumption in particular. To cover this field and for the assessment of environmental benefits associated with the complete replacement of Portland cement, the comparison of CO_2_ emissions per m^3^ of paste is provided in Figure 12. As one can see, the CO_2_ emissions, including all production emissions of designed mixtures, are significantly lowered compared to the Portland cement paste despite high emissions related to sodium silicate production [45,46]. In this example, the high requirements for Portland cement production, especially the calcination of limestone, outweigh the high dosage of sodium silicate [47]. The applied dosage of Na_2_SiO_3_ as a primary activator significantly affects the overall environmental importance and, in some specific cases, can result in higher CO_2_ emissions compared to concrete. Additional benefits are given by the utilization of waste bricks with significantly reduced CO_2_ footprint, neglecting the transportation distances. However, the inclusion of this parameter leads to an unfavorable comparison of the well-established production with a newly emerging alternative that does not have sufficiently optimized processes [48]. It should be mentioned that the CO_2_ profile of the used mixture constituent is also dependent on the energy mix used, as the higher share of renewable energy sources may reduce the contribution of such intensive components [47]. 

**Figure 13:**
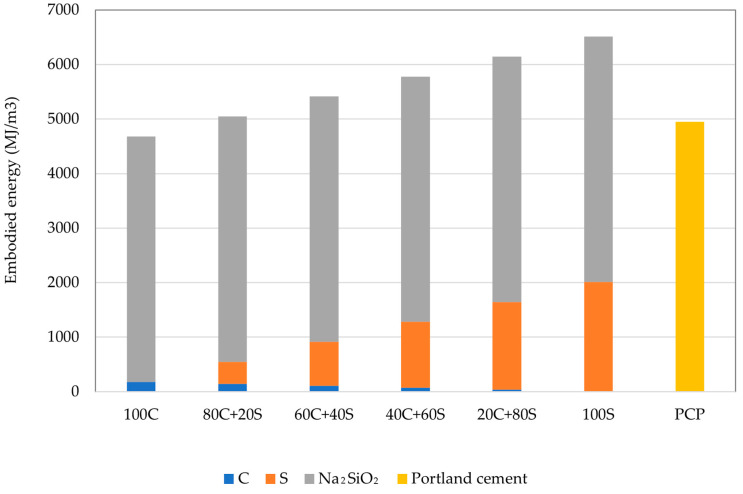
Comparison of embodied energy.

As mentioned in several other papers, the issue of the transfer of burden from one area to another poses a potential risk for overly narrow assessment criteria, as can be visible in Figure 13. According to the Ecoinvent database, the production of sodium silicate requires substantial energy inputs, therefore, damaging the overall environmental footprint compared to traditional binders. The production of 1 ton of 48% Na_2_SiO_3_ consumes approximately 11.2 MJ of non-renewable energy resources and represents about 60 to 90% of all consumed energy for the production of geopolymers. The energy intensiveness of Na_2_SiO_3_ production corresponds to the report published by Fawer et al. [49]. Obtained findings highlight the importance of the replacement of commercial activators and the search for waste sources that can be utilized as sufficient alternatives. On top of that, the reuse of waste sources of alkalis provides significant benefits in the form of avoided production and contribution to the principles of the circular economy [50,51,52]. 

For easy comparison of the environmental impact of designed geopolymers with Portland cement paste, combined indicators by the meaning of the carbon dioxide intensity (*Ci*) and energy intensity (*Ei*) were calculated (see Table 5). It can be seen that all designed mixtures have better CO_2_ efficiency to achieve 1 MPa in compressive strength. However, *Ei* values substantially differ from *Ci* in the sense of efficiency per MPa. Here, only mixtures of 100S, 20C+80S, and 40C+60S attained better scores than PCP, while the rest of the mixtures were worse due to significantly reduced compressive strength performance.

## 4. Conclusions

This study demonstrates the applicability of the blended blast furnace slags and waste brick powder obtained from end-of-life bricks as a precursor for preparing high-performance geopolymer paste. In this sense, six mixtures with different ratios were designed and characterized with a focus on the potential replacement of high-performance concrete. To access the benefits associated with the environmental footprint, a simplified environmental analysis covering the CO_2_ emission and consumption of primary energy was carried out. The major findings can be drawn as follows:

- WBP with lowered content of the amorphous portion compared to GBFS impaired the compressive strength of designed mixtures proportionally. However, even pure WBP can be used as the sole precursor despite the negative results described in the available literature [21]. A high level of compressive strength with WBP content up to 60 wt.% shows a way for the replacement of also high-performance building materials and significant valorization of waste materials.
- The flexural strength was substantially reduced for mixtures with a dominant portion of GBFS to values around 4–5 MPa. These findings correspond to the crack formation as the result of drying shrinkage. On the other hand, this phenomenon was mitigated by the addition of WBP and the dual role of the low-amorphous precursors.
- The employed calorimetry analysis revealed significant differences in the evolution of the hydration heat of both precursors. While the GBFS clearly shows the dissolving, polycondensation, and rearrangements peaks, only the dissolution peak was observed for WBP. Notwithstanding, at a very early age, this peak was significantly more intensive; thus, it may imply the very fact occurrence of all steps (dissolution, polycondensation, rearrangement).
- Despite the fact that geopolymers are investigated mainly with the aim of greening the construction industry, significant environmental savings were obtained only for the CO_2_ emissions. The analysis of the embodied energy revealed a huge impact of using alkaline activators that damaged the potential benefits associated with the utilization of waste or by-products. Described conclusions point to the importance of wider boundary conditions within the environmental assessment and the risk of potentially transferring the environmental burden to another area of protection apart from climate change.

The utilization of WBP requires further research focused on improved reactivity of this precursor in order to attain better mechanical performance. Even pure WBP can be utilized as a precursor for alkali activation, thus close recycling loops can be established to: (i) reduce the volume of the produced waste; (ii) lower the requirement on natural resources. The mechanical strength can be further improved by advanced research of the mix design, for example, by the utilization of the mixture design optimizing tools or the addition of minor content of GBFS according to the desired strength level. This knowledge can be beneficially applied in developing regions where economic development is associated with intensive construction and, consequently, intensified CO_2_ emissions. The advantages of these low-emission materials bring savings in places where the greatest increase in the intensity of CO_2_ production is expected in the near future. 

## Figures and Tables

**Table 1:** Mineralogical composition of used precursors.

		GBFS	WBP
Amorphous phase	-	82	30
Akermanite	Ca_2_Mg(Si_2_O_7_)	12	-
Calcite	CaCO_3_	5	-
Quartz	SiO_2_	1	53
Anorthite	CaAl_2_Si_2_O_8_	-	8
Microcline	KAlSi_3_O_8_	-	4
Orthoclase	KAlSi_3_O_8_	-	1
Muscovite	KAl_2_(AlSi_3_O_10_)(F, OH)_2_	-	4

**Table 2:** Oxide composition of used precursors.

	SiO_2_	CaO	Al_2_O_3_	MgO	MnO	K_2_O	SO_3_	Fe_2_O_3_	Na_2_O	TiO_2_	BaO
GBFS	39.1	38.8	9.8	8.7	0.9	0.7	0.6	0.5	0.4	0.3	0.1
WBP	58.8	6.9	19.6	2.8	-	2.9	0.7	5.7	1.5	0.8	-

**Table 3:** Composition of designed mixtures.

Mixture	WBP (g)	GBFS (g)	Sodium Silicate (M = 1.6) (g)	WA (g)	Water (g)	Precursor Si/Al Ratio
100C	900	0	375	30	0	2.64
80C+20S	720	180	375	30	0	2.87
60C+40S	540	360	375	30	0	3.06
40C+60S	360	180	375	30	5	3.23
20C+80S	180	720	375	30	10	3.38
100S	0	900	375	30	15	3.52

**Table 4:** Bulk and matrix density of designed mixtures.

Mixture	Bulk Density (kg/m^3^)	Matrix Density (kg/m^3^)
100S	2040.9	2496.0
80S+20C	1952.6	2333.0
60S+40S	1902.3	2381.8
40S+60C	1900.8	2490.0
20S+80C	1916.5	2520.2
100C	1905.3	2486.5

**Table 5:** Comparison of CO_2_ and embodied energy efficiency per MPa.

Mixture	*Ci* (kg CO_2_/MPa)	*Ei* (MJ/MPa)
100C	21.83	187.29
80C+20S	15.15	127.81
60C+40S	10.50	87.32
40C+60S	7.91	64.94
20C+80S	7.56	61.46
100S	6.91	55.65
PCP	22.31	76.15

## Data Availability

The data presented in this study are available on request from the corresponding author.

## References

[1] Amran Y.H.M., Alyousef R., Alabduljabbar H., El-Zeadani M. (2020). Clean production and properties of geopolymer concrete; A review. J. Clean. Prod..

[2] Vo D.H., Hwang C.L., Thi K.D.T., Yehualaw M.D., Liao M.C., Chao Y.F. (2021). HPC produced with CDW as a partial replacement for fine and coarse aggregates using the Densified Mixture Design Algorithm (DMDA) method: Mechanical properties and stability in development. Constr. Build. Mater..

[3] Colangelo F., Navarro T.G., Farina I., Petrillo A. (2020). Comparative LCA of concrete with recycled aggregates: A circular economy mindset in Europe. Int. J. Life Cycle Assess..

[4] Robayo-Salazar R.A., Valencia-Saavedra W., de Gutierrez R.M. (2020). Construction and Demolition Waste (CDW) Recycling-As Both Binder and Aggregates-In Alkali-Activated Materials: A Novel Re-Use Concept. Sustainability.

[5] Galan B., Viguri J.R., Cifrian E., Dosal E., Andres A. (2019). Influence of input streams on the construction and demolition waste (CDW) recycling performance of basic and advanced treatment plants. J. Clean. Prod..

[6] Zeng Y.S., Quek S.T., Tang A.P., Zhou X.Y. (2020). Review of Residual Properties of Concrete under Freezing-and-Thawing Loading. ACI Mater. J..

[7] de Brito J., Kurda R. (2021). The past and future of sustainable concrete: A critical review and new strategies on cement-based materials. J. Clean. Prod..

[8] Park S., Wu S., Liu Z.C., Pyo S. (2021). The Role of Supplementary Cementitious Materials (SCMs) in Ultra High Performance Concrete (UHPC): A Review. Materials.

[9] Vejmelkova E., Konakova D., Dolezelova M., Scheinherrova L., Svora P., Keppert M., Reiterman P., Cerny R. (2018). Effect of calcined Czech claystone on the properties of high performance concrete: Microstructure, strength and durability. Constr. Build. Mater..

[10] Yu R., Spiesz P., Brouwers H.J.H. (2015). Development of an eco-friendly Ultra-High Performance Concrete (UHPC) with efficient cement and mineral admixtures uses. Cem. Concr. Compos..

[11] Liu J., Guo R.H. (2018). Applications of Steel Slag Powder and Steel Slag Aggregate in Ultra-High Performance Concrete. Adv. Civ. Eng..

[12] Yang R., Yu R., Shui Z.H., Gao X., Xiao X.G., Zhang X.B., Wang Y.Y., He Y.J. (2019). Low carbon design of an Ultra-High Performance Concrete (UHPC) incorporating phosphorous slag. J. Clean. Prod..

[13] Wu Z.M., Shi C.J., He W. (2017). Comparative study on flexural properties of ultra-high performance concrete with supplementary cementitious materials under different curing regimes. Constr. Build. Mater..

[14] Koci V., Petrikova M., Fort J., Fiala L., Cerny R. (2020). Preparation of self-heating alkali-activated materials using industrial waste products. J. Clean. Prod..

[15] Xiao R., Huang B., Zhou H., Ma Y., Jiang X. (2022). A state-of-the-art review of crushed urban waste glass used in OPC and AAMs (geopolymer): Progress and challenges. Clean. Mater..

[16] Mills J., Mondal P., Wagner N. (2022). Structure-property relationships and state behavior of alkali-activated aluminosilicate gels. Cem. Concr. Res..

[17] Fiala L., Pommer V., Bohm M., Scheinherrova L., Cerny R. (2022). Self-heating alkali activated materials: Microstructure and its effect on electrical, thermal and mechanical properties. Constr. Build. Mater..

[18] Blanco I., D’Angelo A., Viola V., Vertuccio L., Catauro M. (2023). Metakaolin-based geopolymers filled with volcanic fly ashes: FT-IR, thermal characterization, and antibacterial property. Sci. Eng. Compos. Mater..

[19] Fort J., Novotny R., Vejmelkova E., Trnik A., Rovnanikova P., Keppert M., Pommer V., Cerny R. (2019). Characterization of geopolymers prepared using powdered brick. J. Mater. Res. Technol..

[20] Reig L., Tashima M.M., Borrachero M.V., Monzo J., Cheeseman C.R., Paya J. (2013). Properties and microstructure of alkali-activated red clay brick waste. Constr. Build. Mater..

[21] Hwang C.L., Yehualaw M.D., Vo D.H., Huyn T.P. (2019). Development of high-strength alkali-activated pastes containing high volumes of waste brick and ceramic powders. Constr. Build. Mater..

[22] Hassan H.S., Abdel-Gawwad H.A., Vasquez-Garcia S.R., Israde-Alcantara I., Flores-Ramirez N., Rico J.L., Mohammed M.S. (2019). Cleaner production of one-part white geopolymer cement using pre-treated wood biomass ash and diatomite. J. Clean. Prod..

[23] Rakhimova N.R., Rakhimov R.Z. (2015). Alkali-activated cements and mortars based on blast furnace slag and red clay brick waste. Mater. Des..

[24] Luukkonen T., Abdollahnejad Z., Yliniemi J., Kinnunen P., Illikainen M. (2018). One-part alkali-activated materials: A review. Cem. Concr. Res..

[25] Vasic M.V., Terzic A., Radovanovic Z., Radojevic Z., Warr L.N. (2022). Alkali-activated geopolymerization of a low illitic raw clay and waste brick mixture. An alternative to traditional ceramics. Appl. Clay Sci..

[26] Fort J., Cerny R. (2020). Transition to circular economy in the construction industry: Environmental aspects of waste brick recycling scenarios. Waste Manag..

[27] Caldas L.R., Da Gloria M.Y.R., Pittau F., Andreola V.M., Habert G., Toledo R.D. (2021). Environmental impact assessment of wood bio-concretes: Evaluation of the influence of different supplementary cementitious materials. Constr. Build. Mater..

[28] Fort J., Mildner M., Keppert M., Cerny R. (2022). Waste solidified alkalis as activators of aluminosilicate precursors: Functional and environmental evaluation. J. Build. Eng..

[29] Fort J., Vejmelkova E., Konakova D., Alblova N., Cachova M., Keppert M., Rovnanikova P., Cerny R. (2018). Application of waste brick powder in alkali activated aluminosilicates: Functional and environmental aspects. J. Clean. Prod..

[30] Vaclavik V., Ondova M., Dvorsky T., Estokova A., Fabianova M., Gola L. (2020). Sustainability Potential Evaluation of Concrete with Steel Slag Aggregates by the LCA Method. Sustainability.

[31] Palod R., Deo S.V., Ramtekkar G.D. (2019). Utilization of waste from steel and iron industry as replacement of cement in mortars. J. Mater. Cycles Waste Manag..

[32] Najimi M., Ghafoori N., Sharbaf M. (2018). Alkali-activated natural pozzolan/slag mortars: A parametric study. Constr. Build. Mater..

[33] Shen J.L., Li Y., Lin H., Lv J.F., Feng S., Ci J.C. (2022). Early properties and chemical structure analysis of alkali-activated brick geopolymer with varied alkali dosage. J. Build. Eng..

[34] Sedira N., Castro-Gomes J., Magrinho M. (2018). Red clay brick and tungsten mining waste-based alkali-activated binder: Microstructural and mechanical properties. Constr. Build. Mater..

[35] Soultana A., Valouma A., Bartzas G., Komnitsas K. (2019). Properties of Inorganic Polymers Produced from Brick Waste and Metallurgical Slag. Minerals.

[36] Yildirim G., Kul A., Ozcelikci E., Sahmaran M., Aldemir A., Figueira D., Ashour A. (2021). Development of alkali-activated binders from recycled mixed masonry-originated waste. J. Build. Eng..

[37] Li Y., Shen J.L., Lin H., Lv J.F., Feng S., Ci J.C. (2022). Properties and environmental assessment of eco-friendly brick powder geopolymer binders with varied alkali dosage. J. Build. Eng..

[38] Wong C.L., Mo K.H., Yap S.P., Alengaram U.J., Ling T.C. (2018). Potential use of brick waste as alternate concrete-making materials: A review. J. Clean. Prod..

[39] Ye T.H., Xiao J.Z., Duan Z.H., Li S.S. (2022). Geopolymers made of recycled brick and concrete powder—A critical review. Constr. Build. Mater..

[40] Fort J., Vejmelkova E., Keppert M., Rovnanikova P., Bezdicka P., Cerny R. (2020). Alkaline activation of low-reactivity ceramics: Peculiarities induced by the precursors’ dual character. Cem. Concr. Compos..

[41] Koci V., Konakova D., Pommer V., Keppert M., Vejmelkova E., Cerny R. (2021). Exploiting advantages of empirical and optimization approaches to design alkali activated materials in a more efficient way. Constr. Build. Mater..

[42] Lin C.Y., Chen T.A. (2022). Effects of Composition Type and Activator on Fly Ash-Based Alkali Activated Materials. Polymers.

[43] Ke X.Y., Bernal S.A., Provis J.L., Lothenbach B. (2020). Thermodynamic modelling of phase evolution in alkali-activated slag cements exposed to carbon dioxide. Cem. Concr. Res..

[44] Ke X.Y., Duan Y. (2021). Coupling machine learning with thermodynamic modelling to develop a composition-property model for alkali-activated materials. Compos. Part B Eng..

[45] Salas D.A., Ramirez A.D., Ulloa N., Baykara H., Boero A.J. (2018). Life cycle assessment of geopolymer concrete. Constr. Build. Mater..

[46] Bianco I., Tomos B.A., Vinai R. (2021). Analysis of the environmental impacts of alkali-activated concrete produced with waste glass-derived silicate activator-A LCA study. J. Clean. Prod..

[47] Sandanayake M., Law D., Sargent P. (2022). A new framework for assessing the environmental impacts of circular economy friendly soil waste-based geopolymer cements. Build. Environ..

[48] Kavlak G., McNerney J., Trancik J.E. (2018). Evaluating the causes of cost reduction in photovoltaic modules. Energy Policy.

[49] Fawer M., Concannon M., Rieber W. (1999). Life cycle inventories for the production of sodium silicates. Int. J. Life Cycle Assess..

[50] Fort J., Mildner M., Keppert M., Abed M., Cerny R. (2023). Potential of industrial waste as alternative alkaline activator for development of eco-efficient mortars. Case Stud. Constr. Mater..

[51] Vinai R., Soutsos M. (2019). Production of sodium silicate powder from waste glass cullet for alkali activation of alternative binders. Cem. Concr. Res..

[52] Tong K.T., Vinai R., Soutsos M.N. (2018). Use of Vietnamese rice husk ash for the production of sodium silicate as the activator for alkali-activated binders. J. Clean. Prod..

